# Machine learning models to predict surgical case duration compared to current industry standards: scoping review

**DOI:** 10.1093/bjsopen/zrad113

**Published:** 2023-11-03

**Authors:** Christopher Spence, Owais A Shah, Anna Cebula, Keith Tucker, David Sochart, Deiary Kader, Vipin Asopa

**Affiliations:** Academic Surgical Unit, South West London Elective Orthopaedic Centre, Epsom, Surrey, UK; Academic Surgical Unit, South West London Elective Orthopaedic Centre, Epsom, Surrey, UK; Academic Surgical Unit, South West London Elective Orthopaedic Centre, Epsom, Surrey, UK; Academic Surgical Unit, South West London Elective Orthopaedic Centre, Epsom, Surrey, UK; Academic Surgical Unit, South West London Elective Orthopaedic Centre, Epsom, Surrey, UK; Academic Surgical Unit, South West London Elective Orthopaedic Centre, Epsom, Surrey, UK; Academic Surgical Unit, South West London Elective Orthopaedic Centre, Epsom, Surrey, UK

## Abstract

**Background:**

Surgical waiting lists have risen dramatically across the UK as a result of the COVID-19 pandemic. The effective use of operating theatres by optimal scheduling could help mitigate this, but this requires accurate case duration predictions. Current standards for predicting the duration of surgery are inaccurate. Artificial intelligence (AI) offers the potential for greater accuracy in predicting surgical case duration. This study aimed to investigate whether there is evidence to support that AI is more accurate than current industry standards at predicting surgical case duration, with a secondary aim of analysing whether the implementation of the models used produced efficiency savings.

**Method:**

PubMed, Embase, and MEDLINE libraries were searched through to July 2023 to identify appropriate articles. PRISMA extension for scoping reviews and the Arksey and O’Malley framework were followed. Study quality was assessed using a modified version of the reporting guidelines for surgical AI papers by Farrow *et al*. Algorithm performance was reported using evaluation metrics.

**Results:**

The search identified 2593 articles: 14 were suitable for inclusion and 13 reported on the accuracy of AI algorithms against industry standards, with seven demonstrating a statistically significant improvement in prediction accuracy (*P* < 0.05). The larger studies demonstrated the superiority of neural networks over other machine learning techniques. Efficiency savings were identified in a RCT. Significant methodological limitations were identified across most studies.

**Conclusion:**

The studies suggest that machine learning and deep learning models are more accurate at predicting the duration of surgery; however, further research is required to determine the best way to implement this technology.

## Teaser

Waiting lists for elective surgeries are rising in the UK. We conducted a scoping review with the aim to understand if artificial intelligence has the potential to improve the utilization of operating theatre resources through improved scheduling.

## Introduction

Waiting lists for NHS hospital treatment have risen, as they have globally, since the start of the COVID-19 pandemic. This is a result of the postponement of elective surgical procedures^1–3^. In May 2023, 7.5 million patients were waiting for NHS hospital treatment, up from 4.5 million in 2020^4^. The national audit office (NAO) predicts there could be up to 12 million on elective care waiting lists by March 2025^5^.

NHS Improvement reported that optimal theatre utilization could lead to 291 327 further elective operations a year^6^, a 16.8 per cent increase on current levels. This could be achieved by reducing or eliminating inefficiencies such as late starts, early finishes and delays between operations to offset this increase^7^. Additionally, knowledge of the likely duration of a procedure and associated variance would result in further efficiency by improving theatre case scheduling and patient flow, and improving the probability that an operating list would be completed on time^8–10^.

The current industry standards of predicting surgical case duration are based upon a surgeon’s estimate of duration or the mean length of the surgeon’s last ten cases^11^. Both are considered inaccurate because they are limited by a lack of consideration of patient, surgical, nursing, anaesthetic and system factors that may affect how long a surgery lasts^9–13^. Inaccurate predictions lead to both under- and overutilization of theatre time and, as a result, lead to greater costs, fewer patients receiving surgery, reduced patient satisfaction and worsening staff morale^12^. Therefore, being able to better predict surgical case duration will likely improve the efficiency of operating theatre (OT) utilization.

Many studies have used multivariate regression to show improved prediction of surgical duration compared to industry standards^11–14^; however, no single method has achieved widespread acceptance^9,11,15–17^.

The emergence of artificial intelligence (AI) has given rise to a number of studies investigating whether machine learning (ML) and deep learning (DL) algorithms can provide improved predictions compared to multivariate regression and industry-standard methodology^11,16,18–25^. ML algorithms work by extracting knowledge from tabulated data sets, processing them, adjusting their internal parameters (weights) and strengthening associations to increase the model’s accuracy^26^. ‘Learning’ refers to the incremental function optimization that occurs to the weights within the model as it is trained on the data^27^.

**Fig. 1:**
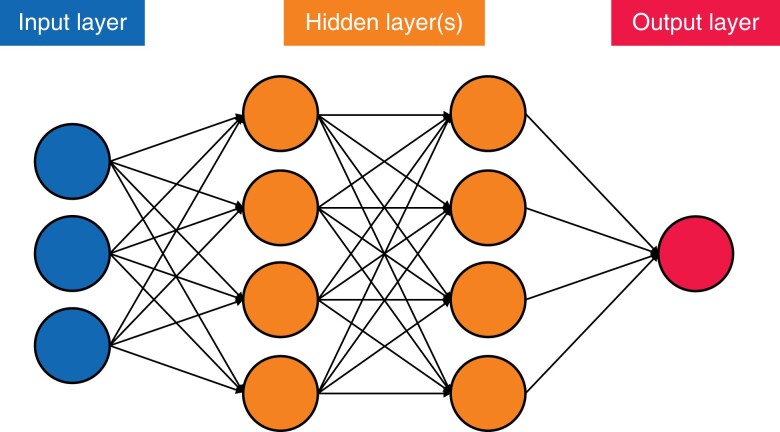
Simplified version of an artificial neural network, demonstrating the principles of connections and weights

DL algorithms, a subset of ML algorithms, are those that are composed of an artificial neural network with three or more layers (*Fig. 1*)^26^. The networks imitate how the human brain functions, which allows these algorithms to ‘learn’ higher-level features of the data that were previously unattainable via traditional ML methods^28^. This ability means that DL algorithms usually outperform other ML techniques^18^.

The increasing availability of larger data sets containing more preoperative variables^18^ and the use of ML/DL data analysis could offer the promise of greater accuracy compared to traditional statistical techniques such as linear regression^29^. Already the increasing availability of large data sets combined with modern computing methods has achieved major successes in other clinical scenarios, such as the detection of intracranial haemorrhages from head CT scan images and the development of clinical decision aids^30,31^.

There are many barriers to the implementation of ML in clinical practice. For example, poor data labelling and categorization, secondary to heterogeneous data sources and poorly designed data structures, hinders the progress of ML in healthcare^32^. Accurate and abundant data are required for these models to be trained on and to allow them to develop accurate predictions. Accurate data are not always available from electronic health records (EHR). Labelling medical data requires knowledge of the field of study, which is time-consuming and expensive when vast quantities of data are required^32^. The aim of this scoping review is to examine the body of work on the utilization of ML/DL algorithms to predict how long a surgical case will last and, as a secondary aim, to establish whether there is evidence for improved efficiency using such methods.

## Methods

The protocol was developed utilizing the PRISMA extension for scoping reviews (*Table S1*)^33^, and Arksey and O’Malley’s five-stage scoping review process^34^ with the adaptations from the Joanna Briggs Institute^35^. The protocol is available upon request.

### Stage 1: identifying the research question

Using the population, intervention, comparator and outcomes of interest framework (*Table 1*), a broad research question was developed: How is AI being utilized to predict surgical case duration and is there a relative improvement in the accuracy of these AI-generated predictions?

**Table 1:** Inclusion, exclusion and population, intervention, comparison and outcome (PICO) criteria for this review

Inclusion criteria	Exclusion criteria
Peer-reviewed articles Articles published in English or with freely available translation Papers discussing the use of different ML models to predict surgical case-time prediction All types of surgery to be included (robotic, arthroscopic, orthopaedics, ENT, etc.)	Studies done on non-human subjects Non-peer-reviewed articles Studies not reported in English or with an easily available translation Abstracts, case studies, case series, review articles, letters, technique papers and book chapters Full-text not available
**PICO criteria**	
Population	Patients undergoing an operation in any surgical speciality
Intervention/exposure	Use of AI-based model to predict case-time duration
Control/comparator	Surgeon estimated/mean of last 10 cases used to predict case-time durations
**Outcome(s)**	
Primary	To analyse the data from different AI models to understand if greater surgical case-time duration prediction is possible with AI models *versus* the current industry standards
Secondary	To establish whether there are efficiency benefits associated with the utilization of ML models in surgical block booking
Tertiary	To understand which models, and with which variables, provide the greatest improvement in case-time prediction

ML, machine learning; ENT, ear, nose and throat; AI, artificial intelligence.

### Stage 2: identifying relevant studies

A systematic search of the literature was performed on 15 November 2021 using both the Healthcare Database Advanced Search (HDAS) searching Medline and EMBASE databases, and the PubMed native search tool, from the start of each respective database to November 2021. The search was most recently updated on 28 July 2023. The set of search terms and Medical Subject Heading (MeSH) terms (*Table S2*) were developed in conjunction with a medical librarian, using both keywords and MeSH terms. A grey literature search was conducted by undertaking a manual search of the reference lists of the included studies and further searches through the Google search engine, Google Scholar, ClinicalTrials.gov and the Cochrane Central Register of Controlled Trials (CENTRAL).

### Stage 3: study selection

Duplicate citations were removed initially; following this, both reviewers (C.S. and O.A.S.) screened the titles and abstracts independently using the inclusion and exclusion criteria outlined in *Table 1*. Full texts of articles that met the criteria were retrieved and reviewed by both C.S. and O.A.S. for inclusion in the study. Disputes were settled upon consultation with a senior author (V.A.) and resolved by group consensus.

### Stage 4: charting the data

All studies that were agreed upon for the final assessment were included in a database. Information was extracted from the articles on study quality, study characteristics, AI model characteristics, the predictive capacity of models, as well as study limitations, conclusions, and recommendations. Data points were extracted and recorded on standardized forms using Microsoft Excel v14.0 (Microsoft Corp., WA, USA).

### Stage 5: collating, summarizing and reporting the results

An assessment of the level of the evidence included was based on the Oxford Centre for Evidence-Based Medicine (OCEBM) criteria^36^. A methodological quality assessment was also performed. Due to the differences between the papers included and traditional surgical research papers, it was decided by the reviewers to create their methodological assessment tool based on work by Farrow *et al.*^37^ (*Table S3*). Due to the heterogeneous nature of the data, it was not possible to perform a meta-analysis on the included studies; therefore, a narrative analysis of the different AI models and their capacity to predict surgical case duration was produced.

All studies used an evaluation metric to assess the model’s capacity to correctly predict surgical case duration when compared to test data. The evaluation metrics used varied between papers but mostly utilized *R*^2^, mean absolute error (MAE), root mean square error (RMSE), mean absolute percentage error (MAPE), continuous ranked probability score (CRPS) and mean square error (MSE). Percentage overage/underage/within was utilized by Bartek *et al*.^18^ in one study. For further details on these metrics see *Table S4*. From the data sets identified within the included studies, the training–validation–test splits were reported as a ratio, as well as specific numbers.

## Results

### Search results and study inclusion

**Fig. 2:**
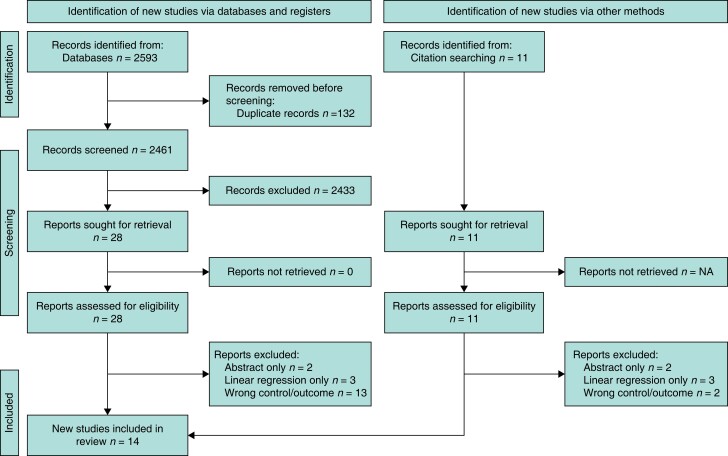
PRISMA diagram demonstrating the process of study selection, from screening to inclusion and the grey literature search (created using the online tool of Haddaway *et al*.^38^)

The systematic literature search described above yielded 2593 articles containing 132 duplicates: 2433 articles were excluded after the title/abstract review and 18 following the full paper review. Following the grey literature search and citation checking, 11 further articles were identified for possible inclusion, of which seven were excluded after a full-text review. Subsequently, 14 articles met the full eligibility criteria. The PRISMA diagram (*Fig. 2*) was created using the online application of Haddaway *et al.*^38^.

### Quality assessment (*Supplementary Table S5*)

Most of the studies included provided their study aims and conflicts of interest; however, Ng *et al.* did not disclose any conflicts of interest and the second author in Tuwatananurak *et al.* is the CEO of the company that developed the ML algorithm^16,25^.

All papers included clear documentation as to the source of their data^11,16,18–25,39–42^. Only one study performed any external validation on their data set; however, Lai *et al.* did discuss their reasons for not performing external validation and Abbas *et al*. internally validated using a national database^19,20,40^. All studies provided information on data extraction and pre-processing^11,16,18–25,39–42^. Three studies did not disclose their management of missing data^22,25,39^. Only one study did not provide clear documentation concerning their approach to model, training, testing, and validation with clearly labelled numbers of samples at each stage^25^. ‘Ground truth’ was outlined in most of the included studies apart from Abbas *et al.*^40^. One study did not provide clear information on the type of models used but did explain that it used supervised learning techniques^24,25^. Only one study provided an example of a power calculation and determined an appropriate sample size^24^.

Only ten of the studies provided clear documentation of the sample’s demographics within their results^11,19,20,22–24,39–42^. All studies included in the paper demonstrated some method of model evaluation^11,16,18–25,39–42^. Thirteen studies demonstrated an explanation of the model with graphs/tables demonstrating which variables had the greatest effect on the model^11,16,18–24,39–42^. Only one study did not discuss limitations^16^. Finally, one study did not discuss barriers to implementation and/or future work^41^.

### Study characteristics

All studies included in the review were published on or after 2017^11,16,18–25,39–42^, and 11 of 14 studies published were in the USA^11,16,18,19,22–25,39^, with three further in Canada^40^, Colombia^21^ and Taiwan^20^ (*Table S6*). Most studies analysed surgeries from multiple specialities; however, six studies focused on individual specialities or surgeries^11,22,39–42^. The size of the original data set varied from 500 up to 302 300^22,40^. Most studies sourced their data from an EHR^11,16,18–25,39,41,42^; Abbas *et al*. utilized the American College of Surgeons’ national surgical and quality improvement database^40^. Most studies were retrospective observational studies; only Stromblad *et al.* was a randomized control trial^24^.

### AI characteristics

The majority of studies included in the review reported purely on how accurately an ML model predicted surgical case duration. One study assessed the efficiency benefits of such a system directly^24^ while a further two explored efficiency savings in their discussion. The number of preoperative variables varied from seven up to >1500^21,25^ (*Table 2*). All studies that reported ‘ground truth’ used the EHR’s recorded ‘wheels in/wheels out’ time for case duration^11,16,18–25,39–42^. Jiao *et al.* were the only study to perform any external validation on their data set; Lai *et al.* discuss that currently, a set does not exist for external validation and that currently, only internal validation is appropriate^19,20^.

**Table 2:** AI model characteristics

Author/Year	Intended use of AI algorithm	Number of preoperative variables utilized as inputs for models	Ground truth label	External validation of data source	Data cleaned prior to analysis	Total no. of patients’ records used	Number of patients used for training/testing	AI/predictive algorithms studied
Ng *et al.*, 2017^16^	Prediction of surgical case-time duration/exploration of homoscedastic *versus* heteroscedastic modelling of data	27	EHR	No	Yes	86 796	∼69 400/∼10 400 (80/8/12 train validation test split)	Linear regression, **MLP** (with Gaussian, Laplace, gamma distributions)
Master *et al.*, 2017^11^	Prediction of surgical case-time duration	8 (+1 with surgeon’s prediction in some models)	EHR	No	Not described	1713	820/893	Single decision tree regressor, random forest regressor, gradient boosted regression trees
Zhao *et al.*, 2018^22^	Prediction of surgical case-time duration	28	EHR	No	Not described	424	424/500 (used entire data set as test)	Multivariable linear regression, ridge regression, lasso regression, random forest, boosted regression tree, **neural network**
Tuwatananurak *et al.*, 2019^25^	Prediction of surgical case-time duration	>1500	EHR	No	Not described	∼15 000	∼15 000/∼990	The proprietary Leap Rail® engine, which uses a combination of supervised learning algorithms
Bartek *et al.*, 2019^18^	Prediction of surgical case-time duration (comparing two models, trained on a procedure-specific model or a surgeon-specific model)	13	EHR	No	Yes	46 986	37 588/9398	Linear regression, extreme gradient boosting (other models used but not reported in paper)
Jiao *et al.*, 2020^23^	Prediction of surgical case-time duration	11	EHR	No	Yes	52 735	42 377/10 358	Decision tree, random forest, gradient boosting decision tree, **mixture density model**
Martinez *et al.*, 2021^21^	Prediction of surgical case-time duration	7	EHR	No	Yes	81 248	∼65 000/∼16 000 (80/20 train:test split)	Linear regression, support vector machines, regression trees, bagged trees
Strömblad *et al.*, 2021^24^	Assessment of benefit on more accurate predictions	Up to 300	EHR	No	Yes	756	605/151 (80/20 train/test split)	Random forest
Lai *et al.*, 2021^20^	Prediction of surgical case-time duration	20	EHR	No	Yes	86 621	∼82 300/4300 (95/5 train/test split)	Linear regression, random forest, extreme gradient boosting
Jiao *et al.*, 2022^19^	Prediction of surgical case-time duration	>16 (not stated clearly)	EHR	Yes	Yes	69 018	59 926/9092 (92.5%/7.5% train/test split)	Bayesian approach, **modular artificial neural network**
Abbas *et al.*, 2022^40^	Prediction of surgical case-time duration and length of stay	32	EHR	No*	Yes	302 300	182 000/57 841/62 459 (Training/validation/test—split by years)	Linear regression, SGD regression, elastic net, linear SVM, KNN, decision tree, random forest, Adaboost, XGBoost, **Scikit-learn MLP**, **PyTorch MLP**
Miller *et al.*, 2022^39^	Prediction of surgical case-time duration	20	EHR	No	Yes	50 888	40 710/10 178 (80:20 training/test split)	CatBoost, XGBoost
Witvoet *et al.*, 2023^41^	Determine how certain variables affected operative time and generate ML predictions for robotic-assisted primary total knee arthroplasty	30	EHR	No	Yes	18 465	14 772/3693 (80:20 training/test split)	CatBoost, **tabNet**
Gabriel *et al.*, 2023^42^	Prediction of surgical case-time duration	9	EHR	No	Yes	3189	2551/638 (80:20)	Linear regression, bagged trees, random forest, XGBoost

Bold indicates DL algorithms. AI, artificial intelligence; EHR, electronic health record; N/A, not applicable; ∼, approximately; SGD, stochastic gradient descent; SVM, support vector machine; KNN, k-nearest neighbour; AdaBoost, adaptive boosting; XGBoost, extreme gradient boosting; MLP, multilayer perceptron; CatBoost, categorical gradient boosting; tabNet, DL algorithm for tabular data. *While they haven’t tested on an external set.

After processing the data using appropriate inclusion/exclusion criteria and removing missing data (not all studies did this), the total number of records used for training and testing varied from 424 up to 302 300^22,40^. There was a large variety in the number of ML algorithms utilized, all of which used supervised learning methodology, but the specific algorithms used were: linear regression, stochastic gradient descent, k-nearest neighbours, single decision tree regressor, random forest regressor, gradient boosted regression trees, extreme gradient boosting, categorical gradient boosting, neural networks, support vector machines, bagged trees, TabNet, multilayer perceptrons and mixture density models.

### Predictive capacity of models

*Table 3* demonstrates the results of each study. Ten of the studies included ‘feature importance’ information (which variable has the greatest impact on the model(s))^11,16,18,20,23,25,39–42^. Three studies reported that the type of procedure was the most important variable on duration^16,20,39^; other studies that reported feature importance commonly demonstrated that expert prediction/scheduled duration, primary surgeon, patient weight and average case-time duration of the latest ten surgeries at the procedure level all had significant impacts on the models they designed^11,18,23,25,41^. Abbas *et al.*^40^ demonstrated that renal failure and transfusions given within 72 h preoperatively were the most important variables; this study only focused on one specific surgery.

**Table 3:** Outcomes of models developed within included studies

Author/Year	Control	Variable(s) with the greatest influence on prediction	Evaluation/comparison metric	AI/predictive algorithms utilized		Performance of AI model(s) *versus* control	AI model more accurate than control (statistically significant)	Conclusion
Ng *et al.*, 2017^16^	Historical averaging, procedure mean	Procedure type	**RMSE**, MAE, NLL	Linear regression,		45.23 *versus* 49.8	Not stated	The study demonstrates the efficacy of machine learning and the heteroscedastic nature of surgical duration data
				MLP (gamma distribution)*		**43.38 *versus* 49.8**	**Yes, *P* = 0.01**	
Master *et al.*, 2017^11^	Historical averaging	Expert prediction/primary surgeon/patient weight	***R* ^2^**, average prediction accuracy by study-derived performance metric	Single decision tree regressor	With EP	0.42 *versus* 0.34	Not stated	New prediction models outclass old models and if used in conjunction with expert opinion outperform expert opinion. Potential for decision support tools to automate OT scheduling
					Without EP	0.28 *versus* 0.34	Not stated	
				Random forest regressor	With EP	0.57 *versus* 0.34	Yes (not stated)	
					Without EP	0.38 *versus* 0.34	Not stated	
				Gradient-boosted regression trees*	With EP	0.61 *versus* 0.34	Yes (Not stated)	
					Without EP	0.44 *versus* 0.34	Not stated	
Zhao *et al.*, 2018^22^	Historical averaging	Not reported	**RMSE**	Multivariable linear regression,		86.8 *versus* 100.4	No (95% confidence intervals)	ML-based predictive models are more accurate than current methods. This will increase the number of accurately booked case durations which may reduce under- and overutilization of OTs
				Ridge regression		82.4 *versus* 100.4	No (95% confidence intervals)	
				Lasso regression		81.3 *versus* 100.4	No (95% confidence intervals)	
				Random forest		81.9 *versus* 100.4	Yes (95% confidence intervals)	
				Boosted regression tree*		80.2 *versus* 100.4	Yes (95% confidence intervals)	
				**Neural network**		**89.6 *versus* 100.4**	**No (95% confidence intervals)**	
Tuwatananurak *et al.*, 2019^25^	Historical averaging	Historical averaging	**Mean absolute difference with interquartile ranges**	The proprietary Leap Rail® engine uses a combination of supervised learning algorithms*		20.0 *versus* 27.0 (for all cases)	Yes, *P* = 0.0001 (for all cases)	Statistically significant improvement of an average of 7 minutes with the LeapRail engine. Post-hoc modelling suggests this could represent a 70% reduction in scheduling inaccuracy
Bartek *et al.*, 2019^18^	Historical averaging and expert predictions	Average case-time duration of latest 10 surgeries at the procedure level	**MAPE**, *R*^2^, percentage overage, percentage underage, percentage within 10%	Linear regression		36% *versus* 30%	Not stated	XGBoost ML models demonstrated the best results *versus* other ML models/current standards. Potential for surgeon-specific ML models to improve scheduling
				Extreme gradient boosting*		26% *versus* 30%	Not stated	
Jiao *et al.*, 2020^23^	Expert prediction primarily (and historical averaging)	Scheduled duration	**CRPS**	Bayesian statistical method		21.2 (min) *versus* 32.1 (min)	Not stated	Demonstrated unstructured hospital data can be used for prediction. Advanced application of ML in this field to potentially inform an intelligent scheduling system
				Decision tree		22.1 (min) *versus* 32.1 (min)	Not stated	
				Random forest		19.6 (min) *versus* 32.1 (mins)	Not stated	
				Gradient boosted decision tree		19.5 (min) *versus versus* 32.1 (min)	Not stated	
				Mixture density network*		**18.1 (min) *versus* 32.1 (min)**	**Not stated**	
Martinez *et al.*, 2021^21^	Historical average and expert prediction	Not reported	**RMSE**	Linear regression		30.84 *versus* 26.09 (*versus* bagged trees)	Not stated	Bagged tree algorithms show an improved overall error rate compared with traditional methods. They recommend research on complementary periods like anaesthesia/cleaning/recovery
				Support vector machine		30.27 *versus* 26.09 (*versus* bagged trees)	Not stated	
				Regression trees		27.94 *versus* 26.09 (*versus* bagged trees)	Not stated	
				Bagged trees*		27.98 *versus* 64.34 (*versus* current standards)	Not stated	
Strömblad *et al.*, 2021^24^	Historical averaging and expert predictions	Not reported	**MAE**	Random forest*		49.5 (mins) *versus* 59.3 (mins) (16.5% improvement)	Yes, *P* = 0.03	Implementation of an ML model significantly improved accuracy in predicting case duration and led to reduced patient wait-time, and reduced pre-surgical length of stay
Lai *et al.*, 2021^19^	Historical average	Procedure type	***R* ^2^**, MAE, percentage overage/underage and within the threshold	Linear regression		0.72 *versus* 0.68	Not stated	The XGB model was superior in predictive performance to the average, regression and random forest models
				Random forest		0.74 *versus* 0.68	Not stated	
				Extreme gradient boosting*		0.77 *versus* 0.68	Not stated	
Jiao *et al.*, 2022^19^	Scheduled duration	Not reported	CRPS	Bayesian approach		20.3 min *versus* 37.0 min	Not stated	They suggest that ML models have a role in informing operational decisions, they are superior to classical scheduling and traditional statistical alternatives. ML may reduce the costs of surgery
				MANN*		**13.8 min *versus* 37.0 min**	***P* < 0.001 (for MANN *versus* Bayesian approach)**	
Abbas *et al.*, 2022^40^	Mean regressor (historical average)	Renal failure, transfusion given within 72 h preoperatively, in/outpatient status, CHF status, presence of disseminated cancer (study performed on only one type of surgery)	**MSE**	Linear regression		0.989 *versus* 1.031	Not stated	The study demonstrated that both deep ML models and conventional ML models were superior to mean regression. However, there was not a significant difference between deep and conventional ML models when looking at accuracy predictions
				SGD regression		1.013 *versus* 1.031	Not stated	
				Elastic Net		0.999 *versus* 1.031	Not stated	
				Linear support vector machine		0.994 *versus* 1.031	Not stated	
				K-nearest neighbours		1.156 *versus* 1.031	Not stated	
				Decision tree		1.032 *versus* 1.031	Not stated	
				Random forest		1.009 *versus* 1.031	Not stated	
				AdaBoost		1.031 *versus* 1.031	Not stated	
				XGBoost		1.003 *versus* 1.031	Not stated	
				**Scikit-learn MLP**		**0.978 *versus* 1.031**	**Not stated**	
				PyTorch MLP*		**0.893 *versus* 1.031**	**Not stated**	
Miller *et al.*, 2022^39^	Historical average	Procedure performed, surgeon, type of case by subspecialty, and surgery case status (day surgery *versus* inpatient)	**RMSE**, MAE	XGBoost		39.3 *versus* 46.3	Yes, *P* < 0.001	Application of a machine learning algorithm to historical otolaryngology case data enabled significant improvement in the prediction of OR case duration. Such methods have the potential to increase case duration accuracy and may result in significant cost savings
				CatBoost*		38.2 *versus* 46.3	Yes, *P* < 0.001	
Witvoet *et al.*, 2023^41^	Historical average	Average surgical time, gender, number of surgeries executed by the surgeon until case date	***R* ^2^**, RMSE, within 5 min %, within 10 min %, within 15 mis %	CatBoost*		0.53 *versus* 0.49	Yes, *P* = 0.003	The ML model developed demonstrated superior accuracy for predicting operative time using hospital, surgeon and patient data compared to historical averages. Hospitals should consider predicting operative times by means of ML algorithms. By doing so they may be able to optimize resource utilization
				**TabNet**		**0.51 *versus* 0.49**	**Yes, *P* = 0.013**	
Gabriel *et al.*, 2023^42^	Historical average and expert prediction	BMI, spine fusion	***R* ^2^**, MAE, RMSE	Linear regression		0.34 *versus* −0.57	Not stated	The use of ensemble learning with patient and procedure specific features (available preoperatively) outperformed the prediction of spine surgery case duration compared to standard predictions. The implementation of ML models presents an alternative pathway to increasing efficiency and enrich patient outcomes
				Random forest		0.76 *versus* −0.57	Not stated	
				Bagged trees		0.76 *versus* −0.57	Not stated	
				XGBoost*		0.77 *versus* −0.57	Not stated	

Bold indicates DL algorithms and which evaluation metric is presented in the sixth column. MANN, modular artificial neural network; RMSE, root mean square error; MAE, mean absolute error; NLL, negative log-likelihood; MAPE, mean absolute percentage error; CRPS, continuous ranked probability score; GBT, gradient boosted decision tree; MDN, mixed density network; OT, operating theatre; ML, machine learning; EP, expert prediction; MSE, mean square error; CHF, congestive heart failure; SGD, stochastic gradient descent; SVM, support vector machine; MLP, multilayer perceptron; XGBoost, extreme gradient boosting; CatBoost, categorical gradient boosting; TabNet, deep neural network for tabular data; AI, artificial intelligence. *Indicates the most accurate model.

**Fig. 3:**
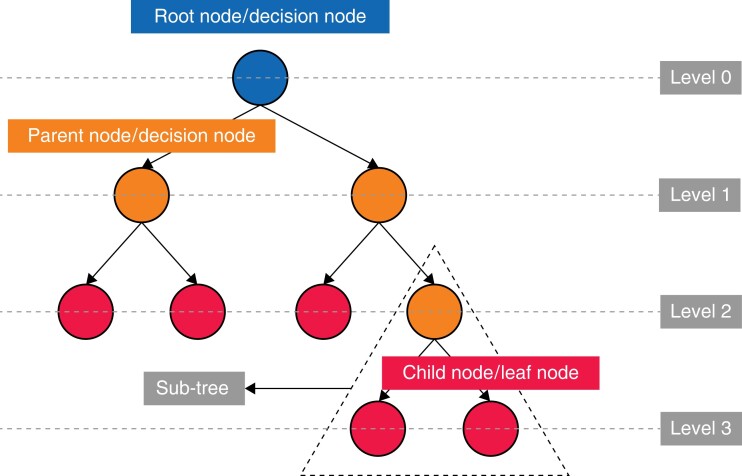
Demonstrating the simple structure of a tree-based algorithm with tree terminology

All studies used several different algorithms to predict the case-time duration from the data provided. Except for the article by Tuwatananurak *et al*., all studies state which algorithms were the most accurate through their testing^25^. Tree-based ML models (*Fig. 3*) account for nine of the 14 best models^11,18,20–22,24,39,41,42^, five of which used some form of gradient boosting^11,18,20,22,39,42^, and one used a bagging method^21^. Six further studies demonstrated improvements in predictive power with DL models (multilayer perceptron, MLP)^16,40^, a mixture density network (MDN)^23^, TabNet^41^ and a modular artificial neural network (MANN)^19^.

### Efficiency savings

Three studies discussed efficiency savings; however, this was too heterogeneous to present in a table. The findings are presented in the discussion.

## Discussion

Among the 14 studies identified, all developed ML algorithms and compared their accuracy to the current industry standards, but only one reported the time efficiency savings that can potentially be achieved by the implementation of such a system^24^. Eleven of the 14 studies were conducted in the USA^11,16,18,19,22–25,39,41,42^, with an overlap of authorship only from Jiao *et al.*^19,23^.

All studies reported the development of an ML model that was more accurate than the current industry standards (historical averaging or surgeon estimation^11,16,18–25,39–42^). Seven of the studies were able to demonstrate that the differences in predictive capability were significant (*P* < 0.05)^16,19,22,24,25,39,41^. This is suggestive of the superior accuracy of ML models in the prediction of surgical case duration. Nine of the studies demonstrated that tree-based ML models were the most accurate as opposed to standard prediction methods and other ML algorithms^11,18,20–22,24,39,41,42^. Tree-based methods are a type of supervised ML model that are popular due to their capacity to handle multifaceted data and their capacity to understand complex or non-linear relationships. These models work by segmenting the predictor space into several simple subsets^43^. There have been advancements in this technique with the development of ‘ensemble learning’ methods including ‘random forests (RF)’^44^, ‘gradient boosting (GBM)’^45^, ‘eXtreme gradient boosting (XGB)’^46^ and CatBoost^47^, which build upon a simple decision tree by aggregating the results of multiple developed trees and combining them. This can provide greater predictive accuracy and more robust models.

Of the studies in this review, only six^16,19,22,23,40,41^ produced models that pertain to ‘deep learning’ techniques. Zhao *et al.*^22^ demonstrated that DL models are not always superior to their ML predecessors; their neural network was the worst-performing model in the study with an RMSE of 89.6 *versus* 80.2 for boosted regression trees (linear regression outperformed the neural network with an RMSE of 86.8)^22^. However, the number of records included to train the models was only 424. Jiao *et al*.^23^ utilized a much larger data set of 52 735 records and showed that their mixture density network (a type of DL model) demonstrated the lowest CRPS of all the models of 18.1 (mins) *versus* 19.5 (mins) for the next closest model, gradient boosted regression trees. In the four studies^16,19,23,40^ that trained DL models using data sets with over 50 000 records, all found that DL models were the most accurate prediction method compared to ML models and the current industry standards, supporting the importance of DL models training on larger data sets.

Jiao *et al.*^19^ developed a unique approach to predicting the duration of surgery. A DL model was developed that continually incorporated preoperative variables as well as intraoperative variables, including vital signs, medications and intraoperative events/stages, called a modular artificial neural network (MANN). The model produced predictions at each fifth percentage of the total duration. This model was compared to the industry standard mean regressor and a simpler model using Bayes theorem. The CRPS (a measure of accuracy) of the MANN improved nearer to the end of the surgery and was statistically significantly better than both the Bayesian method and the mean regressor at all reported time intervals. Jiao *et al.*^19^ further reported that their algorithm could predict impending staff overtime, allowing for better resource management.

The number of variables, features or data sources used within each study varied from seven to over 1500^21,25^. The most common variables were: primary surgeon, historic average surgical duration, the experience of the surgeon, procedure name, the number the procedure lies within the list, type of anaesthesia, duration of the case, patient BMI, patient age, ASA score, patient sex, patient co-morbidities and anaesthesia provider (consultant/junior).

More variables would be expected to allow for more accurate predictions; however, this is not always the case because redundant variables may increase ‘noise’^48,49^. Data science practitioners engage in ‘feature selection’ to identify useful variables and remove those causing noise^48,49^. Another issue is the quality of variable recording and when the data are recorded. For example, ASA may be recorded on the day of surgery, making it unhelpful for planning.

Master *et al*.^11^ found that the ASA score had low importance within their models, suggesting this is because important information within the ASA score may already be coded more clearly within other variables, such as the patient’s weight. Within their model, patient weight had a much larger effect on the outcome across all models compared to the ASA score. This review has shown no significant difference in model accuracy between models using 8 variables compared to over 1500 variables^11,25^.

Two studies predicted the duration of surgery and length of stay for only one procedure, eliminating the ‘procedure performed’ variable^40,41^. The model with the lowest testing MSE for Abbas *et al*. was a PyTorch MLP^40^. The most important features were the presence of renal failure, followed by blood transfusion given within 72 h preoperatively, in-/outpatient status, congestive heart failure status and the presence of disseminated cancer. This confirms that patient factors need to be taken into consideration when defining appropriate data sets for algorithms.

Three studies included in this review discussed efficiency or cost savings directly. Stromblad *et al*.^24^ conducted a prospective interventional RCT with patients randomized to either ML or industry-standard methods to predict the duration of surgery. The resulting predictions were fed into a scheduling workflow for patients undergoing colorectal and gynaecological surgery. Patients assigned to the ML algorithm had a significantly lower MAE of 9.8 min (*P* = 0.03) for duration predictions. Some of the operational benefits noted were that average patient wait times were significantly reduced from 49.4 min to 16.3 min (67.1 per cent improvement) by the utilization of an ML model. The number of large error predictions (MAE > 60 min) was reduced by 8.3 per cent, a significant result as these kinds of large errors can disrupt a day in the OT leading to case cancellation or extended overtime^24^.

Tuwatananurak *et al.*^25^ reported a cumulative reduction of 19 000 min or 70 per cent in scheduling inaccuracy over a 3-month period across the two surgical suites in their medical centre. They identify that the average cost per minute for an operating theatre was estimated between $22 and $133 in the USA at the time of publication^50,51^, highlighting the possible cost savings achievable.

Jiao *et al.* performed a post-hoc analysis of overtime prediction and found that in the 960 cases that overran in the test data, the ML model correctly identified 110 cases more that were going to run overtime compared to the standard prediction method. These cases overran by an average of 154 min. They suggested that if only 10 per cent of overrun cases were identified beforehand, this could avoid 28.2 h of overtime pay by planning for appropriate timely staff handovers during the month sampled^52^. Tuwatananurak and Jiao reported efficiency savings on post-hoc analysis; only Stromblad *et al*. confirmed the benefits of ML through a prospective study.

Many of the included studies were of poor quality. Eleven of 14 (75 per cent) studies^11,16,18,21–25,39,41,42^ did not discuss or report any external validation practices on their models, limiting the algorithm’s use outside of the host institution. Only one of the included studies performed external validation^19^. Jiao *et al.*^19^ generated a database from a different but local hospital to test and train their algorithm on. However, Abbas *et al.*^40^ utilized data from an American national database and internally validated it by splitting the data according to years, providing generalizability to the whole country.

Although both techniques improve the generalization of the algorithms, they require testing on wider data. Three of 14 studies^22,25,39^ did not clearly state how missing data were managed; mismanagement of missing data can lead to reduced statistical power and create bias within the results following ML analysis^53–55^. Tuwatananurak *et al.*^25^ did not disclose how the model(s) were trained, tested or validated. This information is required to reproduce study findings and for critical appraisal. Four studies^16,18,21,25^ did not include baseline reporting of sample characteristics to allow the reader to confirm whether randomization or splitting of the data was appropriate^48,49^.

Several studies^11,22,24^ used small data sets (<1000 records used) to train their algorithms, leaving the algorithms susceptible to overfitting (aligning too closely to statistical noise, rendering predictions on new data poor) and being inadequately powered. It is clear that larger data sets are necessary, including national databases; however, this requires further work on improving the connectivity and accessibility of such data. Standardization could allow different researchers to access large data sets from multiple centres, improving algorithm development and reducing the limitation of only accessing data from one or two data sites/centres.

Generally, there is the challenge of comparing the performance of algorithms due to methodological heterogeneity; evaluation metrics varied between studies. Most studies lacked external validation, meaning that these algorithms may not be applicable to other institutions. Studies often lacked detail on the technical aspects of the ML models used. Finally, some articles, unpublished or not indexed, may have been missed.

The authors note that issues of implementing ML models into clinical practice in the papers identified are sparse. Some issues are highlighted relating to real-time data pipelines^19^ and how categorical data are encoded into the models^11^, and Strömblad *et al.* discuss ensuring data availability weeks prior to surgery being a requirement for a prospective model^24^. The authors recommend that implementation issues are considered in future studies and that implementation research could be utilized to enhance study protocol design^56–58^.

The models demonstrated in this review produced predictions retrospectively or one day in advance. Implementing AI-based case duration predictions in surgical centres requires models to predict surgical duration weeks in advance.

This is one of the first studies to utilize Farrow *et al.*’s^37^ proposed standardized reporting of predictive ML research and the proposed TRIPOD-AI^59^ statement to improve the conduct, reproducibility and comparability of further surgical-AI research.

Despite the development of ML algorithms, it is remarkable that only 14 suitable studies of low-level evidence, published since 2017, have been identified for inclusion in this review^26,60^. This is likely due to multiple barriers including poor-quality data (collection and recording)^61,62^, lack of standardization and the use of multiple systems which lack compatible formats/interoperability^51,52^. The industry should be encouraged to develop an open application programming interface (API) standard.

Furthermore, the skills required to create and implement a successful healthcare-based ML model require input from computer scientists, surgeons, anaesthetists and medical managers; unless there is a dedicated research unit, it may be difficult to maintain cooperation between these diverse teams^63^.

Obtaining ethical approval provides a further barrier that many tech-based solutions encounter when attempting to acquire the large quantities of data that are required for ML/DL models^64^. Numerous ethical issues include accountability for errors that arise from the use of an ML algorithm^65^. Who is to blame, the physician, the algorithm designers or the institution purchasing the technology^65^?

Implementing AI solutions requires addressing both human factors and technological factors. Technological factors included better data collection, extraction from current EHR systems^25^, categorization and pipelines^66^. The human factors include people having capabilities, opportunities and the motivation to ensure that such systems function adequately. Allocating resources to close these gaps is vital to the successful implementation of AI solutions in healthcare systems^64^. ML is only one facet of improving theatre utilization. Other important factors include developing protocols to reduce the turnover time between patients^67^. Other benefits may be realized through the implementation of ML models into electronic theatre scheduling pathways. These include the introduction of new technologies and staff training, which may encourage further improvements related to efficiency^64^.

To integrate these modern solutions the NHS needs to demand and drive standardization of our technological resources. Software developers must create APIs that allow ML models to access the data they require. New standards should be developed regarding the implementation and development of open-source APIs that allow for secure data extractions and interoperability between different software packages. To support this drive and deliver the changes successfully, the NHS must also consider human factors such as staff education^64^.

These studies suggest that DL and ML models can be used to predict surgical case duration and they will perform more accurately than the current industry standards. There is early evidence to suggest these improvements in accuracy will lead to efficiency and cost benefits, but more work is needed to identify the best way to implement these models.

## Supplementary Material

zrad113_Supplementary_Data

## Data Availability

The authors confirm that the data supporting the findings of this study are available within the article and its Supplementary Materials. Other resources can be made available upon request.
