# Supplementary material for: Machine learning models to predict surgical case duration compared to current industry standards: scoping review
Source: BJS Open. 2023 Nov 3;7(6):zrad113. doi: 10.1093/bjsopen/zrad113 (PMC10630142; doi:10.1093/bjsopen/zrad113)
Supplement: zrad113_Supplementary_Data [file zrad113_supplementary_data.docx]

**A scoping review of the use of machine learning models to predict surgical case duration compared against the current industry standards**

**Supplementary Materials - Index**

| **Supplementary Tables** |  |
| --- | --- |
| PRISMA Scoping Review Checklist  Search strategy | *page 2*  *page 4* |
| Explanation of quality assessment  Glossary of terms  Quality assessment  Study characteristics | *page 7*  *page 9*  *page 10*  *page 12* |
| **References** | *page 14* |

| **Supplementary Figures and Tables**  **Supplementary Table 1:** Preferred Reporting Items for Systematic reviews and Meta-Analyses extension for Scoping Reviews (PRISMA-ScR) Checklist^1^   \| **SECTION** \| **ITEM** \| **PRISMA-ScR CHECKLIST ITEM** \| **REPORTED ON PAGE #** \| \| --- \| --- \| --- \| --- \| \| **TITLE** \| \| \| \| \| Title \| 1 \| Identify the report as a scoping review. \| #1 \| \| **ABSTRACT** \| \| \| \| \| Structured summary \| 2 \| Provide a structured summary that includes (as applicable): background, objectives, eligibility criteria, sources of evidence, charting methods, results, and conclusions that relate to the review questions and objectives. \| #2-3 \| \| **INTRODUCTION** \| \| \| \| \| Rationale \| 3 \| Describe the rationale for the review in the context of what is already known. Explain why the review questions/objectives lend themselves to a scoping review approach. \| #4-6 \| \| Objectives \| 4 \| Provide an explicit statement of the questions and objectives being addressed with reference to their key elements (e.g., population or participants, concepts, and context) or other relevant key elements used to conceptualize the review questions and/or objectives. \| #4-6 \| \| **METHODS** \| \| \| \| \| Protocol and registration \| 5 \| Indicate whether a review protocol exists; state if and where it can be accessed (e.g., a Web address); and if available, provide registration information, including the registration number. \| #6 \| \| Eligibility criteria \| 6 \| Specify characteristics of the sources of evidence used as eligibility criteria (e.g., years considered, language, and publication status), and provide a rationale. \| #6-7 & Table 1 \| \| Information sources* \| 7 \| Describe all information sources in the search (e.g., databases with dates of coverage and contact with authors to identify additional sources), as well as the date the most recent search was executed. \| #7 \| \| Search \| 8 \| Present the full electronic search strategy for at least 1 database, including any limits used, such that it could be repeated. \| #7 and Table 2 \| \| Selection of sources of evidence† \| 9 \| State the process for selecting sources of evidence (i.e., screening and eligibility) included in the scoping review. \| #7 \| \| Data charting process‡ \| 10 \| Describe the methods of charting data from the included sources of evidence (e.g., calibrated forms or forms that have been tested by the team before their use, and whether data charting was done independently or in duplicate) and any processes for obtaining and confirming data from investigators. \| #7 \| \| Data items \| 11 \| List and define all variables for which data were sought and any assumptions and simplifications made. \| #7-8 \| \| Critical appraisal of individual sources of evidence§ \| 12 \| If done, provide a rationale for conducting a critical appraisal of included sources of evidence; describe the methods used and how this information was used in any data synthesis (if appropriate). \| #8 \| \| Synthesis of results \| 13 \| Describe the methods of handling and summarizing the data that were charted. \| #8 \| \| **RESULTS** \| \| \| \| \| Selection of sources of evidence \| 14 \| Give numbers of sources of evidence screened, assessed for eligibility, and included in the review, with reasons for exclusions at each stage, ideally using a flow diagram. \| #8-9 and Figure 2 \| \| Characteristics of sources of evidence \| 15 \| For each source of evidence, present characteristics for which data were charted and provide the citations. \| #10 and Table 2 \| \| Critical appraisal within sources of evidence \| 16 \| If done, present data on critical appraisal of included sources of evidence (see item 12). \| #9-10 and Supplementary Table 5 \| \| Results of individual sources of evidence \| 17 \| For each included source of evidence, present the relevant data that were charted that relate to the review questions and objectives. \| #10-12 & Table 2/3 \| \| Synthesis of results \| 18 \| Summarize and/or present the charting results as they relate to the review questions and objectives. \| #10-12 & Table 2/3 \| \| **DISCUSSION** \| \| \| \| \| Summary of evidence \| 19 \| Summarize the main results (including an overview of concepts, themes, and types of evidence available), link to the review questions and objectives, and consider the relevance to key groups. \| #13-20 \| \| Limitations \| 20 \| Discuss the limitations of the scoping review process. \| #18 \| \| Conclusions \| 21 \| Provide a general interpretation of the results with respect to the review questions and objectives, as well as potential implications and/or next steps. \| #20 \| \| **FUNDING** \| \| \| \| \| Funding \| 22 \| Describe sources of funding for the included sources of evidence, as well as sources of funding for the scoping review. Describe the role of the funders of the scoping review. \| #1 \|   **Supplementary Table 2 –** Search strategies   \| **Number** \| **Database** \| **Search details** \| \| --- \| --- \| --- \| \| 1 \| Medline & Embase \| *"ARTIFICIAL INTELLIGENCE"/ \| \| 2 \| Medline & Embase \| (Computational intelligence).ti,ab \| \| 3 \| Medline & Embase \| (Predictive modelling).ti,ab \| \| 4 \| Medline & Embase \| (Predictive modeling).ti,ab \| \| 5 \| Medline & Embase \| (Supervised learning).ti,ab \| \| 6 \| Medline & Embase \| (Artificial Intelligence).ti,ab \| \| 7 \| Medline & Embase \| (Deep learning).ti,ab \| \| 8 \| Medline & Embase \| (Machine Learning).ti,ab \| \| 9 \| Medline & Embase \| (Machine Algorithm).ti,ab \| \| 12 \| Medline & Embase \| *”OPERATIVE TIME”/ - Medline  “OPERATIVE DURATION”/ - Embase \| \| 13 \| Medline & Embase \| *”INTRAOPERATIVE PERIOD”/ \| \| 14 \| Medline & Embase \| (“Operating time”).ti,ab \| \| 15 \| Medline & Embase \| (“Operative time”).ti,ab \| \| 16 \| Medline & Embase \| (“Surgical time*”).ti,ab \| \| 17 \| Medline & Embase \| (“Surgery time*”).ti,ab \| \| 18 \| Medline & Embase \| (“Operative duration”).ti,ab \| \| 19 \| Medline & Embase \| (“Case-time”).ti,ab \| \| 20 \| Medline & Embase \| (“case time”).ti, ab \| \| 21 \| Medline & Embase \| (“Surgery duration”).ti,ab \| \| 22 \| Medline & Embase \| (“Surgical duration”).ti,ab \| \| 23 \| Medline & Embase \| (12 OR 13 OR 14 OR 15 OR 16 OR 17 OR 18 OR 19 OR 20 OR 21 OR 22) \| \| 24 \| Medline & Embase \| (1 OR 2 OR 3 OR 4 OR 5 OR 6 OR 7 OR 8 OR 9) \| \| 25 \| Medline & Embase \| (23 AND 39) \| \| 1 \| Pubmed \| ((((((((((("artificial intelligence"[MeSH Terms]) OR (Computational Intelligence[Title/Abstract])) OR (predictive modelling[Title/Abstract])) OR (predictive modeling[Title/Abstract])) OR (Supervised Learning[Title/Abstract])) OR (Deep learning[Title/Abstract])) OR (Machine learning[Title/Abstract])) OR (Machine algorithm[Title/Abstract]))) \| \| 2 \| Pubmed \| ((((((((((((operative time[MeSH Terms]) OR (intraoperative period [MeSH Terms])) OR (Operating time[Title/Abstract])) OR (Operative time[Title/Abstract])) OR ("Surgical time"[Title/Abstract])) OR ("Surgery time"[Title/Abstract])) OR ("Operative duration"[Title/Abstract])) OR ("case-time"[Title/Abstract])) OR ("case time"[Title/Abstract]) ) OR ("Surgery Duration"[Title/Abstract])) OR (Surgical Duration[Title/Abstract]))))) \| \| 3 \| Pubmed \| #1 AND #2 \| |  |
| --- | --- | --- | --- | --- | --- | --- | --- | --- | --- | --- | --- | --- | --- | --- | --- | --- | --- | --- | --- | --- | --- | --- | --- | --- | --- | --- | --- | --- | --- | --- | --- | --- | --- | --- | --- | --- | --- | --- | --- | --- | --- | --- | --- | --- | --- | --- | --- | --- | --- | --- | --- | --- | --- | --- | --- | --- | --- | --- | --- | --- | --- | --- | --- | --- | --- | --- | --- | --- | --- | --- | --- | --- | --- | --- | --- | --- | --- | --- | --- | --- | --- | --- | --- | --- | --- | --- | --- | --- | --- | --- | --- | --- | --- | --- | --- | --- | --- | --- | --- | --- | --- | --- | --- | --- | --- | --- | --- | --- | --- | --- | --- | --- | --- | --- | --- | --- | --- | --- | --- | --- | --- | --- | --- | --- | --- | --- | --- | --- | --- | --- | --- | --- | --- | --- | --- | --- | --- | --- | --- | --- | --- | --- | --- | --- | --- | --- | --- | --- | --- | --- | --- | --- | --- | --- | --- | --- | --- | --- | --- | --- | --- | --- | --- | --- | --- | --- | --- | --- | --- | --- | --- | --- | --- | --- | --- | --- | --- | --- | --- | --- | --- | --- | --- | --- | --- | --- | --- | --- | --- | --- | --- | --- | --- | --- | --- | --- | --- | --- | --- | --- | --- | --- |

|  |  |
| --- | --- |
| **Supplementary Table 3:** Explaining how the quality assessment was conducted for each of the criteria. Modified from Farrow et al^24^ |  |

| **Item** | **Explanation** |
| --- | --- |
| **Introduction** |  |
| Study aims | The aim of the study should be reported in the introduction. This should include the intended users of the ML algorithm. |
| Disclosure statement | A disclosure statement should be included and reported. |
| **Methods** |  |
| Data Source | Clear documentation of the data source is required including the dates involved. |
| Indication of data quality | Identification of whether external validation of the data source has been performed, and its accuracy compared to a gold standard; reference. |
| Methods for data extraction and pre-processing | How the data was prepared for input to the ML model should be explained, e.g. how imaging data were  standardized. The approach will depend on the type and quantities of data available. |
| Assessment and management of  missing data | The assessments performed to identify the amount and types of missing data present should be documented,  including how missing data were handled in the analyses. |
| Method of model training, testing,  and validation | The method of performing model training, testing, and validation must be included, with documentation of the number of samples involved in each stage. |
| Ground truth | Clearly defined “True” answer including how this “True” answer was derived from the source data |
| Output type | The type of ML model used should be specified. This includes whether the model was pre-trained and/or  supervised, and used a probabilistic versus deterministic approach. |
| Sample size calculation | The methods by which the sample size was obtained should be described, as well as the techniques used to ensure the sample is sufficient. |
| **Results** |  |
| Sample reporting including  baseline characteristics | Studies should report the total number of participants and their baseline characteristics. |
| Model evaluation | The AUC metric or C-statistic for classification models should be presented, ideally with 95% CIs. A confusion matrix should also be specified. If using a classification model compared to a gold standard reference, then the Kappa index (Cohen’s Kappa) should also be presented. For probabilistic models, the Brier score or log loss should also be identified. |
| Model explanation | Clinical models should include relevant analyses that allow for an understanding of the key clinical parameters  involved in decision-making, e.g. global variable importance plots for prediction models, or heat maps in diagnostic radiology. |
| **Conclusions** |  |
| Interpretation of the model in clinical practice | How the model should be interpreted (and used if appropriate) with regard to clinical practice should be  discussed. |
| Limitations | Limitations of the methods and their relevance to clinical practice should be discussed. |
| Barriers to implementation/future  research | Any barriers to implementation within the clinical practice should be discussed with the identification of how these may  be overcome in future. |

**Supplementary Table 4 –** Table describing the evaluation metrics utilised within the included studies.

| R^2^ | R^2^ is a statistical measure of how close the data is fitted to the line of regression. It is defined as the percentage of the response variable variation that is explained by the independent variable(s) (R^2^ = 1- (explained variation / total variation)). This is often represented between 1 and 0, with 1 demonstrating a perfect model that perfectly explains the variance in the dependent variable^2,3^. |
| --- | --- |
| Mean Absolute Error (MAE) | MAE is defined as the average of the magnitude of the errors in a set of predictions, without considering their direction. This means the values can vary from zero to infinity, with a lower MAE indicating closer predictions^4,5^. |
| Mean Square Error (MSE) | The average squared difference between the predicted and observed values (residuals). If a model were to have no error the MSE would equal 0. The greater the difference between the predicted and observed values the greater the MSE. |
| Root Mean Square Error (RMSE) | RMSE is the square root of the mean of the square of all the errors. It is commonly used for numerical predictions. In simpler terms it represents the standard deviation of the residuals (the difference between the predicted outcome and the actual outcome), providing a measure of how closely the data points fit a line of best fit. Translating this into evaluating models it means a smaller RMSE represents a closer fitting model^6^. |
| Mean Absolute Percentage Error (MAPE) | MAPE represents the mean of the absolute percentage errors of predictions. The metric is easier to understand as the result is demonstrated as a percentage, with a smaller MAPE indicating closer predictions^7^. |
| Continuous Ranked Probability Score (CRPS) | CRPS is the mean square error of the predicted cumulative density function (CDF) against the true CDF (CDF is a method used to describe the distribution of random variables across a whole dataset). The ideal score is 0 for this function, making its interpretation similar to that of RMSE and MAE^8,9^. |
| Percentage underage/overage/within | Percentage underage/overage/within uses the study’s own assumptions on what is an acceptable error. Bartek et al. define a less than 10% difference in prediction as an acceptable prediction error. Underage refers to those cases that lasted at least 10% less than the predicted value and overage represents the cases that last more than 10% longer than the predictions^10^. |

**Supplementary Table 5**: Assessment of the methodological quality of the papers included in the review (2 = Reported and adequate, 1 = Reported but inadequate, 0 = not reported, 1* Not reported or performed but discussed and justified, ** Internal validation on a nationwide data source (dataset split into years for training/validation/testing), *** data on duration obtained from national database)

| **Author/Year** | **Study aim(s)** | **Disclosure statement** | **Data Source** | **Indication of data quality** | **Methods for data extraction and reprocessing** | **Assessment and management of missing data** | **Method of model training, testing, and validation** | **Ground truth** | **Output type** | **Sample size calculation** | **Sample reporting including baseline characteristics** | **Model Evaluation** | **Model explanation** | **Interpretation of the model in clinical practice** | **Limitations** | **Barriers to implementation/future research** |
| --- | --- | --- | --- | --- | --- | --- | --- | --- | --- | --- | --- | --- | --- | --- | --- | --- |
| **Ng et al, 2017**^11^ | 2 | 0 | 2 | 0 | 2 | 2 | 2 | 2 | 2 | 1 | 0 | 2 | 2 | 2 | 0 | 2 |
| **Master et al, 2017**^12^ | 2 | 2 | 2 | 0 | 2 | 2 | 2 | 2 | 2 | 1 | 2 | 2 | 2 | 0 | 2 | 2 |
| **Zhao et al, 2018**^13^ | 2 | 2 | 2 | 0 | 2 | 0 | 2 | 2 | 2 | 1 | 2 | 2 | 2 | 2 | 2 | 2 |
| **Tuwatana-nurak et al, 2019**^15^ | 2 | 2 | 2 | 0 | 2 | 0 | 1 | 2 | 1 | 1 | 0 | 2 | 0 | 2 | 2 | 2 |
| **Bartek et al, 2019**^16^ | 2 | 2 | 2 | 0 | 2 | 2 | 2 | 2 | 2 | 0 | 0 | 2 | 2 | 2 | 2 | 2 |
| **Jiao et al, 2020**^9^ | 2 | 2 | 2 | 0 | 2 | 2 | 2 | 2 | 2 | 0 | 2 | 2 | 2 | 2 | 2 | 2 |
| **Martinez et al, 2021**^14^ | 2 | 2 | 2 | 0 | 2 | 2 | 2 | 2 | 2 | 0 | 0 | 2 | 2 | 2 | 2 | 2 |
| **Strömblad et al, 2021**^17^ | 1 | 2 | 2 | 0 | 2 | 2 | 2 | 2 | 2 | 2 | 2 | 2 | 2 | 2 | 2 | 2 |
| **Lai et al, 2021**^18^ | 2 | 2 | 2 | 1* | 2 | 2 | 2 | 2 | 2 | 0 | 2 | 2 | 2 | 2 | 2 | 2 |
| **Jiao et al, 2022**^19^ | 2 | 2 | 2 | 2 | 2 | 2 | 2 | 2 | 2 | 0 | 2 | 2 | 0 | 2 | 2 | 2 |
| **Abbas et al, 2022**^20^ | 2 | 2 | 2 | 1** | 2 | 2 | 2 | 0*** | 2 | 0 | 2 | 2 | 2 | 2 | 2 | 2 |
| **Miller et al, 2022**^21^ | 2 | 2 | 2 | 0 | 2 | 0 | 2 | 2 | 2 | 0 | 2 | 2 | 2 | 2 | 2 | 2 |
| **Witvoet et al, 2023**^22^ | 2 | 2 | 2 | 0 | 2 | 2 | 2 | 2 | 2 | 0 | 2 | 2 | 2 | 2 | 2 | 0 |
| **Gabriel et al. 2023**^23^ | 2 | 2 | 2 | 0 | 2 | 2 | 2 | 2 | 2 | 0 | 2 | 2 | 2 | 2 | 2 | 2 |

**Supplementary Table 6 -** Study Characteristics

| **Author/Year** | **Journal/Conference** | **Location of study** | **Study period** | **No. of patients / size of original dataset** | **Surgical Specialties** | **Source Of Data** | **Study Design** | **Level Of Evidence** |
| --- | --- | --- | --- | --- | --- | --- | --- | --- |
| Ng et al, 2017^11^ | Proceedings of the 2nd Machine Learning for Healthcare Conference | USA (San Diego) | 2014-2016 | 107,755 | Non-excluded | EHR from single large hospital | Retrospective observational | III |
| Master et al, 2017^12^ | International Journal of Data Science and Analytics | USA (Palo Alto) | May 2015 – Jan 2016 | 4898 | Paediatric (10 most common procedures) | EHR from single large hospital | Retrospective observational | III |
| Zhao et al, 2018^13^ | Journal of Medical Systems | USA (San Diego) | Jan 2014 – June 2017 | 500 (randomly selected sample from whole dataset) | Robot-assisted surgery (12 most common procedures) | EHR from single large hospital | Retrospective observational | III |
| Tuwatananurak et al, 2019^15^ | Journal of Medical Systems | USA (Solano County) | Jan 2018 – March 2018 | 15,000 | Non excluded (procedures excluded based on inadequate data) | EHR from single large hospital | Retrospective observational | III |
| Bartek et al, 2019^16^ | Journal of the American College of Surgeons | USA (Seattle) | Jan 2014 – Dec 2017 | 46,986 | Excluded if procedure not in main theatres | EHR from single large hospital | Retrospective observational | III |
| Jiao et al, 2020^9^ | Journal of the American Medical Informatics Association | USA (Saint Louis) | Jan 2017 – Dec 2017 | 53, 783 | Non-Excluded | EHR from single large hospital | Retrospective observational | III |
| Martinez et al, 2021^14^ | Computer Methods and Programs in Biomedicine | Colombia (Bogota) | Dec 2004 – April 2019 | 206,587 | Non-excluded | EHR from single large hospital | Retrospective observational | III |
| Strömblad et al, 2021^17^ | JAMA Surgery | USA (New York City) | April 2018 – June 2018 | 869 | Gynaecology/  Colorectal cancer surgeries | EHR from single large hospital | Randomized  clinical trial | II |
| Lai et al, 2021^18^ | Journal of Anaesthesia and Clinical Research | Taiwan (Taichung) | Jan 2017 – Dec 2019 | 179,510 | Non-excluded | EHR from single large hospital | Retrospective observational | III |
| Jiao et al, 2022^19^ | British Journal of Anaesthesia | USA (Washington) | Mar 2019 – Oct 2019 | 79, 752 | Non-excluded | EHR from 8 hospital within the BJC HealthCare System | Retrospective observational | III |
| Abbas et al, 2022^20^ | International Journal of Medical Informatics | Canada (Toronto) | Jan 2014 – Dec 2019 | 302,300 | Orthopaedics (Total Knee Arthroplasty) | American College of Surgeon’s – National surgical and quality improvement database | Retrospective observational | III |
| Miller et al, 2022^21^ | Otolaryngology -- head and neck surgery | USA (Boston) | Jan 2016 – Aug 2020 | 50,888 | Otolaryngology | EHR from single large hospital | Retrospective observational | III |
| Witvoet et al, 2023^22^ | Knee Surgery, Sports Traumatology, Arthroscopy: | USA (Boston) | Jan 2007 – Dec 2020 | 22,356 | Orthopaedic (Robotic surgery) | Stryker health cloud | Retrospective observational | III |
| Gabriel et al, 2023^23^ | JMIR Perioperative Medicine | USA (San Diego) | Jan 2018 – Sept 2021 | 3523 | Orthopaedic (Spinal surgery) | EHR from single large hospital | Retrospective observational | III |

**References**

1. Tricco AC, Lillie E, Zarin W, O’Brien KK, Colquhoun H, Levac D, et al. PRISMA Extension for Scoping Reviews (PRISMA-ScR): Checklist and Explanation. Ann Intern Med [Internet]. 2018 Oct 2 [cited 2022 May 19];169(7):467–73. Available from: https://pubmed.ncbi.nlm.nih.gov/30178033/

2. Regression Analysis: How Do I Interpret R-squared and Assess the Goodness-of-Fit? [Internet]. [cited 2022 Jan 26]. Available from: https://blog.minitab.com/en/adventures-in-statistics-2/regression-analysis-how-do-i-interpret-r-squared-and-assess-the-goodness-of-fit

3. Georga EI, Fotiadis DI, Tigas SK. Nonlinear Models of Glucose Concentration. Personalized Predictive Modeling in Type 1 Diabetes. 2018;131–51.

4. MAE and RMSE — Which Metric is Better? | by JJ | Human in a Machine World | Medium [Internet]. [cited 2022 Jan 26]. Available from: https://medium.com/human-in-a-machine-world/mae-and-rmse-which-metric-is-better-e60ac3bde13d

5. Fürnkranz J, Chan PK, Craw S, Sammut C, Uther W, Ratnaparkhi A, et al. Mean Absolute Error. Encyclopedia of Machine Learning [Internet]. 2011 [cited 2022 Jan 26];652–652. Available from: https://link.springer.com/referenceworkentry/10.1007/978-0-387-30164-8_525

6. RMSE: Root Mean Square Error - Statistics How To [Internet]. [cited 2022 Jan 26]. Available from: https://www.statisticshowto.com/probability-and-statistics/regression-analysis/rmse-root-mean-square-error/

7. Swamidass PM, editor. MAPE (mean absolute percentage error)MEAN ABSOLUTE PERCENTAGE ERROR (MAPE). In: Encyclopedia of Production and Manufacturing Management [Internet]. Boston, MA: Springer US; 2000. p. 462. Available from: https://doi.org/10.1007/1-4020-0612-8_580

8. What Is A Cumulative Distribution Function? | by Emmett Boudreau | Towards Data Science [Internet]. [cited 2022 Jan 26]. Available from: https://towardsdatascience.com/what-is-a-cumulative-distribution-function-2e0540ec2a60

9. Jiao Y, Sharma A, Abdallah A Ben, Maddox TM, Kannampallil T. Probabilistic forecasting of surgical case duration using machine learning: Model development and validation. Journal of the American Medical Informatics Association. 2020 Dec 1;27(12):1885–93.

10. Bartek MA, Saxena RC, Solomon S, Fong CT, Behara LD, Venigandla R, et al. Improving Operating Room Efficiency: Machine Learning Approach to Predict Case-Time Duration. In: Journal of the American College of Surgeons. Elsevier Inc.; 2019. p. 346-354.e3.

11. Ng N, Gabriel RA, McAuley J, Elkan C, Lipton ZC. Predicting Surgery Duration with Neural Heteroscedastic Regression. 2017 Feb 17 [cited 2021 Oct 21]; Available from: https://arxiv.org/abs/1702.05386v3

12. Master N, Zhou Z, Miller D, Scheinker D, Bambos N, Glynn P. Improving predictions of pediatric surgical durations with supervised learning. Int J Data Sci Anal. 2017 Aug 1;4(1):35–52.

13. Zhao B, Waterman RS, Urman RD, Gabriel RA. A Machine Learning Approach to Predicting Case Duration for Robot-Assisted Surgery. J Med Syst. 2019 Feb 1;43(2).

14. Martinez O, Martinez C, Parra CA, Rugeles S, Suarez DR. Machine learning for surgical time prediction. Comput Methods Programs Biomed. 2021 Sep 1;208.

15. Tuwatananurak JP, Zadeh S, Xu X, Vacanti JA, Fulton WR, Ehrenfeld JM, et al. Machine Learning Can Improve Estimation of Surgical Case Duration: A Pilot Study. J Med Syst. 2019 Mar 1;43(3).

16. Bartek MA, Saxena RC, Solomon S, Fong CT, Behara LD, Venigandla R, et al. Improving Operating Room Efficiency: Machine Learning Approach to Predict Case-Time Duration. In: Journal of the American College of Surgeons. Elsevier Inc.; 2019. p. 346-354.e3.

17. Strömblad CT, Baxter-King RG, Meisami A, Yee SJ, Levine MR, Ostrovsky A, et al. Effect of a Predictive Model on Planned Surgical Duration Accuracy, Patient Wait Time, and Use of Presurgical Resources: A Randomized Clinical Trial. JAMA Surg. 2021 Apr 1;156(4):315–21.

18. Lai J, Huang JY, Liu SC, Cho DY, Yu J. Improving and Interpreting Surgical Case Duration Prediction with Machine Learning Methodology. J Anesth Clin Res. 2021;12:998.

19. Jiao Y, Xue B, Lu C, Avidan MS, Kannampallil T. Continuous real-time prediction of surgical case duration using a modular artificial neural network. Br J Anaesth. 2022 Jan;

20. Abbas A, Mosseri J, Lex JR, Toor J, Ravi B, Khalil EB, et al. Machine learning using preoperative patient factors can predict duration of surgery and length of stay for total knee arthroplasty. Int J Med Inform [Internet]. 2022 [cited 2022 Jun 8];158(2):1386–5056. Available from: https://doi.org/10.1016/j.ijmedinf.2021.104670

21. Miller LE, Goedicke W, Crowson MG, Rathi VK, Naunheim MR, Agarwala A v. Using Machine Learning to Predict Operating Room Case Duration: A Case Study in Otolaryngology: https://doi.org/101177/01945998221076480 [Internet]. 2022 Feb 8 [cited 2022 Jun 8]; Available from: https://journals.sagepub.com/doi/10.1177/01945998221076480?url_ver=Z39.88-2003&rfr_id=ori%3Arid%3Acrossref.org&rfr_dat=cr_pub++0pubmed

22. Witvoet S, de Massari D, Shi S, Chen AF. Leveraging large, real-world data through machine-learning to increase efficiency in robotic-assisted total knee arthroplasty. Knee Surg Sports Traumatol Arthrosc [Internet]. 2023;31(8):3160–71. Available from: http://ovidsp.ovid.com/ovidweb.cgi?T=JS&PAGE=reference&D=mesx&NEWS=N&AN=36650339

23. Gabriel RA, Harjai B, Simpson S, Du AL, Tully L, George O, et al. An Ensemble Learning Approach to Improving Prediction of Case Duration for Spine Surgery : Algorithm Development and Validation Corresponding Author : 6:1–13.

24. Farrow L, Zhong M, Ashcroft GP, Anderson L, Meek RMD. Interpretation and reporting of predictive or diagnostic machine-learning research in Trauma & Orthopaedics. Bone Joint J. 2021;103(12):1754–8.
